# Tibio-Femoral Contact Force Distribution is Not the Only Factor Governing Pivot Location after Total Knee Arthroplasty

**DOI:** 10.1038/s41598-018-37189-z

**Published:** 2019-01-17

**Authors:** A. Trepczynski, I. Kutzner, P. Schütz, J. Dymke, R. List, P. von Roth, P. Moewis, G. Bergmann, W. R. Taylor, G. N. Duda

**Affiliations:** 1 Julius Wolff Institute and Center for Musculoskeletal Surgery, Charité – Universitätsmedizin Berlin, corporate member of Freie Universität Berlin, Humboldt-Universität zu Berlin, and Berlin Institute of Health, Berlin, Germany; 2 Institute for Biomechanics, ETH Zürich, Zürich, Switzerland

## Abstract

Total knee arthroplasty aims to mimic the natural knee kinematics by optimizing implant geometry, but it is not clear how loading relates to tibio-femoral anterior-posterior translation or internal-external pivoting. We hypothesised that the point of pivot in the transverse plane is governed by the location of the highest axial force. Tibio-femoral loading was measured using an instrumented tibial component in six total knee arthroplasty patients (aged 65–80y, 5–7y post-op) during 5–6 squat repetitions, while knee kinematics were captured using a mobile video-fluoroscope. In the range of congruent tibio-femoral contact the medial femoral condyle remained approximately static while the lateral condyle translated posteriorly by 4.1 mm (median). Beyond the congruent range, the medial and lateral condyle motions both abruptly changed to anterior sliding by 4.6 mm, and 2.6 mm respectively. On average, both the axial loading and pivot position were more medial near extension, and transferred to the lateral side in flexion. However, no consistent relationship between pivoting and load distribution was found across all patients throughout flexion, with R^2^ values ranging from 0.00 to 0.65. Tibio-femoral kinematics is not related to the load distribution alone: medial loading of the knee does not necessarily imply a medial pivot location.

## Introduction

Natural tibio-femoral (TF) kinematics during a load bearing high flexion activity have been described as a femoral roll-back relative to the tibia with increasing knee flexion, mostly on the lateral side, resulting in an external rotation of the femur around a medial pivot^1–3^. During gait, the TF contact force is thought to act predominantly through the medial compartment^4–7^, where most early osteoarthritis (OA) has been observed^8,9^. Natural medial and lateral tibial compartments are not symmetrical, and differences in their shape and size suggest an optimization for medial pivoting^10,11^, while many total knee arthroplasty (TKA) designs use a more symmetrical tibial plateau geometry. Joint kinematics after TKA is often different from natural knees, involving a “paradoxical” anterior translation of the femur and lateral pivoting with increasing flexion^12–15^. The altered kinematics can lead to increased patello-femoral contact forces and potentially to problems of the extensor mechanism, often resulting in anterior knee pain^16^, but also to altered loading patterns in the surrounding soft tissues^17^. The “paradoxical” anterior sliding also increases the amount of relative motion between the contact surfaces, and thus the energy dissipated by friction, plausibly leading to more wear^18^. Modern TKA designs aim to restore the natural knee kinematics by optimizing implant geometry. For example, medial pivot designs attempt to combine a more constrained ball-and-socket type joint medially with a lateral side that allows more anterior-posterior (AP) motion, in order to enforce medial pivoting^19,20^. Abrupt changes of the femoral condyle curvature have been proposed as an initiator of anterior sliding, which can be reduced by introducing a gradually changing femoral radius^21,22^.

However, until recently it was not clear how the dynamic loading of the knee is related to AP motion and pivoting *in vivo*, primarily because obtaining *in vivo* joint contact forces is difficult. For this study, we were able to measure a unique TKA cohort with instrumented implants allowing direct measurements of the *in vivo* TF loading^23^, combined with a mobile fluoroscope that enabled tracking of the internal TF kinematics throughout complete cycles of activities of daily living^24,25^. Within this comparison of kinetics and kinematics, we focused on the squat activity, which is similar to everyday activities like sitting down and rising from a seated position, but provides a consistently high joint loading throughout a large range of knee flexion. Therefore, the squat is regularly used to investigate the loaded kinematics of the knee^26–29^. The aim of this study was to evaluate whether the medio-lateral (ML) pivot location (in the transverse plane) coincides with the ML distribution of the TF contact force. The motivation for this question comes from: (1) the fact that predominant loading and pivoting have been observed to coincide on the medial side, (2) the mechanical consideration that a higher axial force should constrain the AP-motion more (through higher friction and grade resistance on the inlay slopes), and (3) the fact that the investigated design is symmetrical, so the geometry by itself cannot explain the observed off-centre pivoting. Consequently, we hypothesized that the pivot is localized in the compartment with the higher axial force.

## Materials and Methods

**Figure 1:**
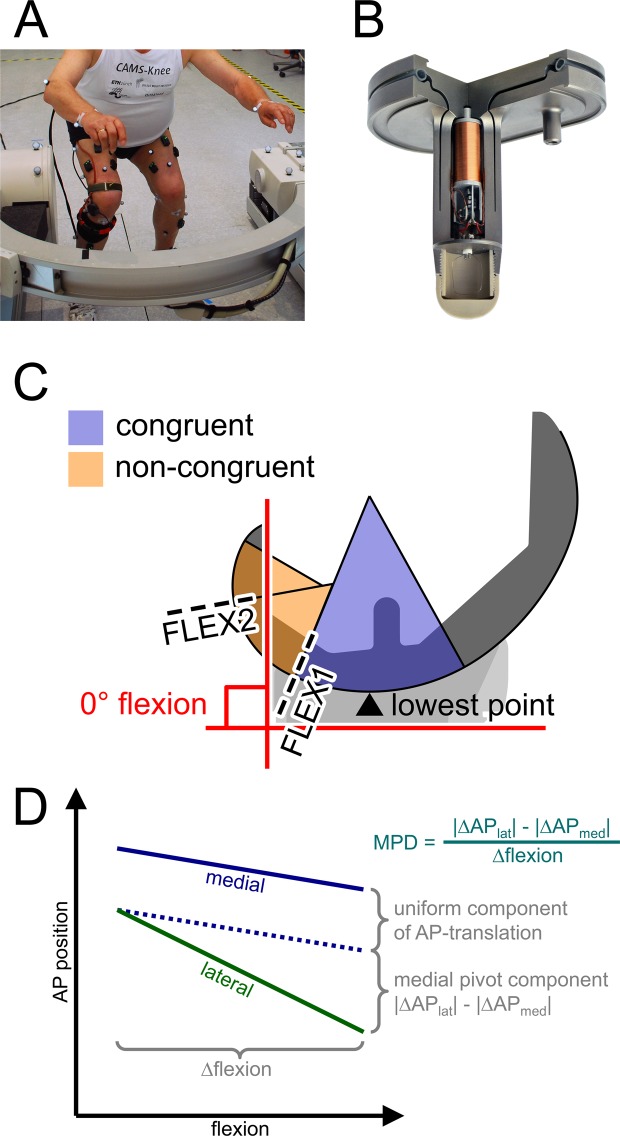
(**A**) The experimental setup with one of the subjects performing a squat within the mobile C-arm of the mobile fluoroscope. (**B**) The instrumented tibial component used to measure tibio-femoral contact loading *in vivo*. (**C**) The definition of flexion (angle between tibial implant’s base plate and most posterior cut plane of the femoral implant. Flexion ranges separated by FLEX1. Lowest femoral point (closest point to the tibial base plate). (**D**) Explanation of the medial pivot delta (MPD), which was used to quantify the pivoting of the femur relative to the tibia, based on the differential AP motion of the lowest femoral points.

This study uses data from the CAMS-Knee measurements, which have been described in more detail previously^25^. Hence, only a brief summary of the measurements is provided here: Six TKA patients: 5 male, 1 female, aged 77(65–80) [median, range] years, 5–7y post-op, mass 88(63–95) kg, height 174(165–175) cm, implanted with a posterior cruciate sacrificing (PCS) INNEX-FIXUC implant (Zimmer, Switzerland), performed 5–6 repetitions of a squat activity (Fig. 1A). All subjects provided written informed consent to participation in the investigations and the publication of their images. The study was approved by the local ethics committees (ETH Ethikkommission: EK 2013-N-90, Charité Ethikkommission: EA4/069/06). All investigations were performed in accordance with relevant guidelines/regulations. TF contact loading was measured using an instrumented tibial component (Fig. 1B)^23^, while TF kinematics were captured synchronously using a mobile video-fluoroscope^24^. The medial and lateral axial force components F_med_, F_lat_ were derived from the *in vivo* measurement as reported by Kutzner *et al*.^4^, where the medial force ratio (MFR) was defined as:

$${\rm{MFR}}={{\rm{F}}}_{{\rm{med}}}/{(F}_{{\rm{med}}}+{{\rm{F}}}_{{\rm{lat}}})$$

where F_med_ + F_lat_ represents the total axial contact force. The anterior force ratio (AFR) was defined as the ratio of the anterior contact force component F_ant_ to the axial component F_axial_:

$${\rm{AFR}}={{\rm{F}}}_{{\rm{ant}}}/{{\rm{F}}}_{{\rm{axial}}}$$

The relative 3D positions of the implant components were reconstructed from the fluoroscopic images. An earlier investigation with the same system and a similar TKA design reported the rotational/translational errors of the 3D reconstruction as 0.15°/0.3 mm in plane, and 0.25°/1 mm out of plane^24^. Since the AP-motion used to quantify the pivoting in this study was mostly in plane with the image intensifier, the accuracy of the reported results can be expected to be similar to the in plane accuracy. The relative implant positions were then used to determine flexion and the AP position of the femoral condyles based on their lowest points relative to the tibial plateau (Fig. 1C)^21^. Flexion was defined based on the relative orientation of the implant components, which in some patients resulted in negative flexion values during standing, due to the placement of the components relative to the bones. The components were considered at 0° flexion when the plane of the posterior femoral cut was perpendicular to the tibial base plate (Fig. 1C). The congruent flexion range was defined as containing all flexions below the value, at which the femoral curvature at the lowest femoral point changes from a radius almost matching the tibial inlay radius, to a smaller radius (FLEX1). Flexions greater than FLEX1 constituted the non-congruent range, which also included flexions beyond a second radius change at FLEX2 (Fig. 1C). The tibial inlay and the femoral component of the INNEX system are both medio-laterally symmetrical in the areas relevant for their interaction. To investigate how the knee pivots axially during flexion and extension of the knee, only time points at which the knee flexion was changing with at least 5° per second (dynamic phases) were considered. These dynamic phases covered at least 98.7% of the original flexion range of a patient and consisted of 3755 time points in total for all patients. First order splines were fitted by the least squares method into the kinematic and loading data as functions of flexion for each patient individually. The spline knots were placed at ~20° wide intervals, with one of the knots was adjusted to FLEX1 to specifically match the transition from the femoral curvature radius that is congruent with the tibial inlay geometry to a smaller radius. The root mean square errors of the spline fits are given in Table 1. The AP position of the medial and lateral lowest point was plotted as a function of flexion, and as a line connecting them (lowest line) in an axial view of the tibial inlay. To indicate the corresponding medio-lateral load distribution in the axial view, a point around which moments of F_med_ and F_lat_ in the frontal plane balance was introduced on each lowest line. The subsequent analyses of AP translation and pivoting were based on the fitted spline values. The gradients of the spline segments of the AP position were interpreted as AP movement with respect to flexion (∆AP/∆flex) associated with the corresponding flexion interval. To quantify the pivoting for each interval, the medial pivot delta (MPD) was defined as:

$${\rm{MPD}}=|{{\rm{\Delta }}\mathrm{AP}}_{{\rm{lat}}}/{\rm{\Delta }}\mathrm{flex}|-|{{\rm{\Delta }}\mathrm{AP}}_{{\rm{med}}}/{\rm{\Delta }}\mathrm{flex}|{\rm{.}}$$

Here, positive MPD values indicate medial pivoting, negative values lateral pivoting, while a MPD of zero corresponds to either a central pivot location or a complete lack of axial rotation (Fig. 1D). Differences in MFR and MFD between the congruent and non-congruent flexion range were investigated statistically using the Mann-Whitney-U test on the combined MFR data all patients, and MPD based on per patient spline fits. The correlations between MFR with MPD and MFR with AFR across all 20°-flexion intervals were investigated using linear regression using the lme4 R-package^30,31^.

**Table 1:** The RMSEs for the spline fits into all time points of a patient that showed movement (flexing or extending).

Patient	spline fit RMSE	
	AP position (med., lat.) [mm]	AP force (flex., ext.) [N]
K1L	0.50, 0.67	43, 31
K2L	0.88, 0.77	27, 24
K3R	0.62, 0.64	21, 21
K5R	0.33, 0.50	24, 32
K7L	0.83, 0.92	12, 23
K8L	0.47, 0.50	33, 30

## Results

### Congruent versus non-congruent contact range

**Figure 2:**
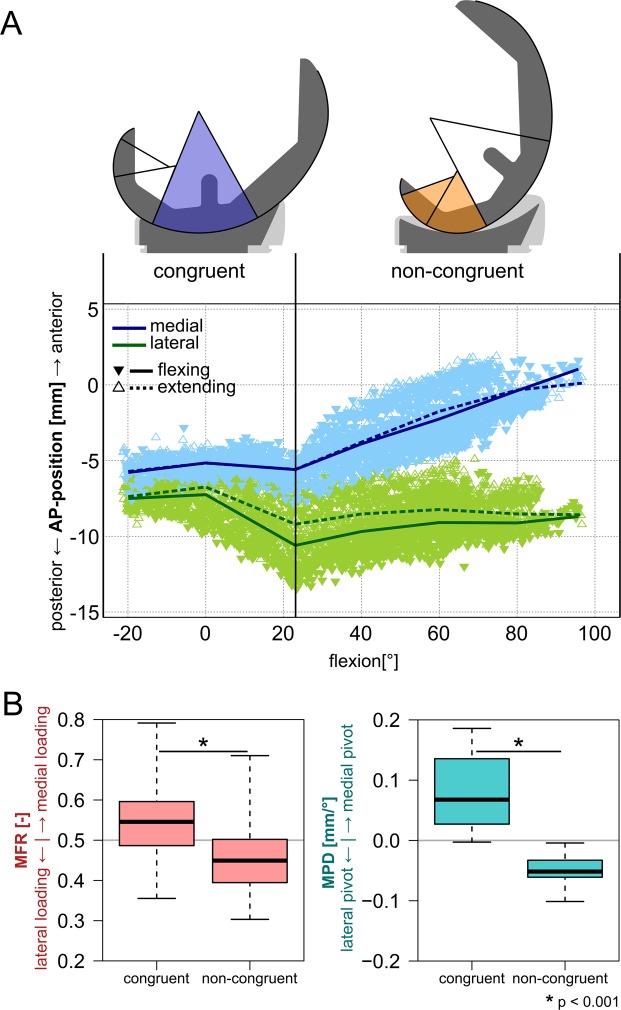
(**A**) Anterior-posterior position (AP) of the lowest femoral points relative to the tibial plateau for all patients combined. (**B**) Medio-lateral loading distribution (MFR) and pivoting (MPD) for the congruent and non-congruent flexion ranges across all patients.

**Figure 3:**
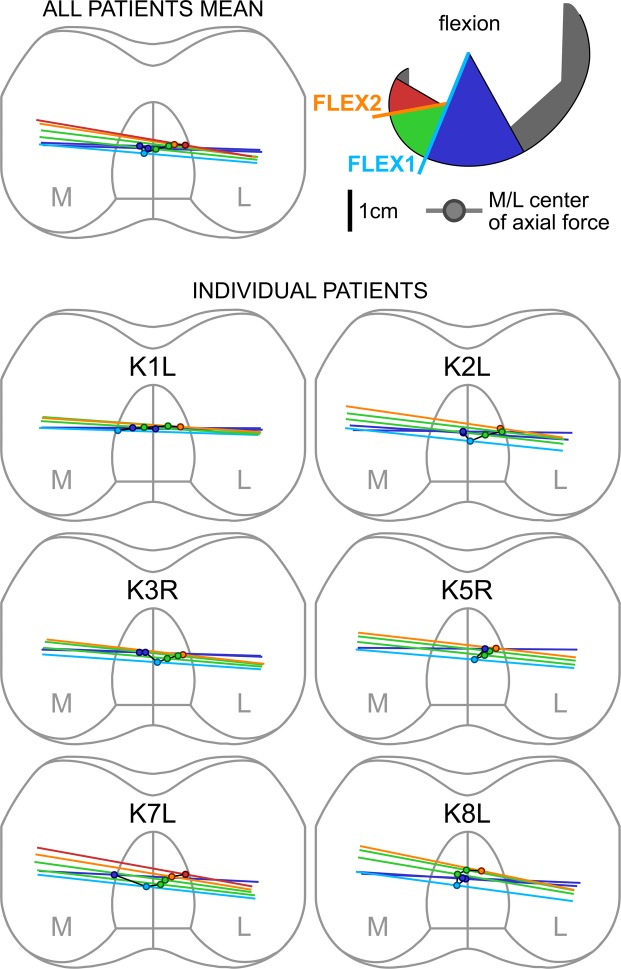
The positions of the line connecting the lowest femoral points (lowest line) at different flexions (colour scale), based on the spline fits into the combined data from flexing and extending. The coloured dots represent the points on each lowest line around which the moments from the medial and lateral axial force components would balance in the frontal plane (its offset from the centre indicates the side of more axial loading).

During the squat maximal knee flexion angles of 71° to 97° were reached, while the external rotation of the femur relative to the tibia increased continuously from between −1° and 4° at minimum flexion to between 3° and 13° at maximum flexion (Table 2). The AP movement of the lowest femoral points had two distinct phases, with an abrupt change at the end of the congruent range, at FLEX1 (Figs 2A and 3). From full extension to FLEX1, the medial side showed little AP-movement, while the lateral condyle points moved posteriorly by 4.1 (1.1 to 4.4) mm (median, range). From FLEX1 to maximal flexion achieved by the patient during the squat, both femoral condyles translated anteriorly relative to the tibia, but the extent of this “paradoxical” sliding was greater on the medial side: 4.6 (2.0 to 6.6), than on the lateral side: 2.6 (0.9 to 3.1). On average, there were significant differences between the congruent and non-congruent contact range in terms of MFR and MPD (p < 0.001). Both, the loading and pivot positions were predominantly located medially for the congruent range with MFR: 0.55 (0.36 to 0.79) and MPD: 0.07 (0.0 to 0.19) mm/°, but transferred to the lateral side in the non-congruent range with MFR: 0.45 (0.30 to 0.71) and MPD: −0.05 (−0.10 to 0.0) mm/° (Fig. 2B).

**Table 2:** Range of flexion based on the relative component orientations and the corresponding external femoral rotations based on the lowest femoral points.

Patient	Pre-OP knee score	Squat: knee kinematics			
		Min. flexion [°] median (range)	Max. flexion [°] median (range)	Ext. fem. rot. at min. flexion [°] median (range)	Ext. fem. rot. at max. flexion [°]median (range)
K1L	65	−12 (−15, −8)	67 (62, 73)	0 (−1, 3)	4 (3, 5)
K2L	75	−21 (−22, −18)	68 (50, 80)	1 (−1, 2)	8 (4, 8)
K3R	63	−16 (−17, −15)	63 (58, 71)	1 (0, 2)	6 (6, 8)
K5R	90	7 (−3, 8)	84 (81, 86)	2 (1, 2)	7 (7, 8)
K7L	70	−2 (−7, 0)	87 (76, 97)	4 (2, 4)	11 (9, 12)
K8L	95	−21 (−21, −20)	73 (71, 76)	2 (2, 4)	12 (12, 13)

### Relationships throughout flexion

When MFR and MPD were compared for all 20°-flexion intervals using linear regression, there was no consistent relationship across all patients and joint motion directions, with R^2^ values ranging from 0.00 to 0.65 (Table 3). Notably, the patient with the lowest peak axial loads (K7L) showed the strongest correlations between MFR and MPD, with R^2^ values ranging from 0.56 and 0.65 during flexing and extending respectively. The AFR also failed to show a consistent relationship to MPD, but was in some cases a better predictor of MPD, than MFR.

**Table 3:** The coefficients of determination from linear regression of each patient.

Patient	R^2^: MPD ~ MFR		R^2^: MPD ~ AFR	
	Flexing	Extending	Flexing	Extending
K1L	0.02	0.33	0.14	0.02
K2L	0.18	0.33	0.26	0.32
K3R	0.57	0.07	0.40	(0.00)
K5R	0.26	(0.00)	0.26	(0.00)
K7L	0.56	0.65	0.66	0.30
K8L	0.23	0.09	0.50	0.23

The MFR (medial force ratio), and the AFR (anterior force ratio) were used as predictors of the MPD (medial pivot delta). Values in brackets indicate regressions that were not significantly different from an intercept only model (p > 0.05).

**Figure 4:**
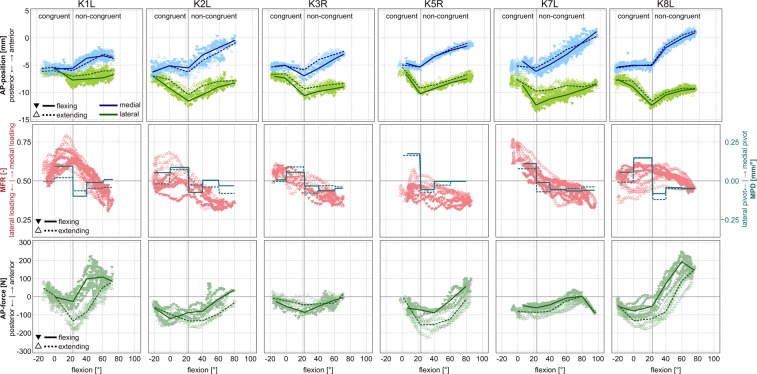
Kinematics and loading as function of flexion for all 6 patients. TOP: The anterior-posterior (AP) movement of the lowest femoral points. CENTER: The medial force ratio (MFR) and the medial pivot delta (MPD). BOTTOM: The anterior-posterior (AP) component of the tibio-femoral contact force acting on the tibia.

The three patients with highest axial loads (K1L, K5R, K8L), also showed greater ranges of AP force throughout flexion, and greater differences in AP force between flexing vs. extending, than the other three patients (Fig. 4, Table 4). Patients K1L and K8L also tended to transfer the pivot more abruptly from the medial to the lateral side, immediately after the end of the congruent contact range, which didn’t match the axial loading distribution in that phase (Fig. 3, Table 4).

**Table 4:** Loading data of individual patients.

Patient	Trial peak loads	Δ Flexing-extending		
	Axial force [N] median (range)	Min. AP - force [N] median (range)	Max. AP - force [N] median (range)	AP-force [N] median (range)
K1L	2484 (2230, 2695)	−146 (−181, −109)	178 (76, 220)	89 (9, 143)
K2L	2247 (1921, 2760)	−160 (−168, −138)	35 (−30, 71)	53 (−15, 67)
K3R	2012 (1639, 2025)	−103 (−120, −61)	25 (9, 81)	−19 (−36, 1)
K5R	3033 (2870, 3085)	−164 (−224, −127)	83 (33, 104)	78 (10, 88)
K7L	1380 (1348, 1418)	−97 (−130, −89)	−2 (−14, 38)	26 (−2, 31)
K8L	2440 (2394, 2664)	−127 (−176, −106)	221 (145, 246)	61 (24, 129)

The peak axial & AP-loading (anterior: positive, posterior: negative) given as median and range across the repetitions of each patient, based on all data points. The difference in AP-loading between flexing and extending, based the difference in splines fitted to each direction of joint motion, as median and range across the 20° flexion intervals.

## Discussion

An understanding of the relationship between TKA loading and kinematics is essential for restoring knee function and lays the foundations for implants to reproduce natural kinematics. However, so far little is known about the interplay between kinematics and kinetics *in vivo*. The external loading of the joint and the geometry of the articulating surfaces are known to interact in a non-trivial manner, resulting in local kinematics and contact loading that is hard to predict. Direct synchronized measurements of the internal knee kinematics and loading are very rare; therefore, the cohort investigated in this study presents to our knowledge the first empirical evidence on whether the load distribution among the compartments affects their differential motion. Given the inconsistent relationships between the ML force distribution and the pivot position across patients and 20°-flexion intervals, our hypothesis should be rejected. The results show that in some TKA patients, the pivot location was not correlated to the ML load distribution throughout flexion, so that a predominantly medial knee loading does not necessarily imply a medial pivot. Other factors affecting kinematics, possibly including the absolute level of loading or neuro-motor control patterns (e.g. co-contraction), seem to dominate pivoting in some patients.

Within this small but unique group of TKA patients, an external femoral rotation was found with increasing flexion. However, the amount of external rotation was smaller than that reported for natural knees^3^. More importantly, the AP motion of the femoral implant condyles differed substantially from observations in natural knees^1–3^: Instead of a continuous lateral “roll back” and medial pivoting with increasing flexion, the TKA knees examined in our study exhibited only a short phase of lateral condyle posterior movement together with medial pivoting, followed by an abrupt change to “paradoxical” anterior translation of both condyles. Due the change in direction, the total range of lateral AP movement observed in this study was also smaller than observed in natural knees (~5 mm vs. ~10 mm)^29,32^, where the tibial contact surface is much flatter^33^, and thus allows more roll-back than the congruent inlay employed here. The change in AP kinematics coincided with the change of the femoral radius at FLEX1 in this specific implant design (INNEX), which supports previous findings connecting curvature discontinuities to initiation of anterior sliding^22^, and to a shift of the functional axis^34^. Femoral external rotation continued at higher flexion angles, when the condyles were no longer congruent, as demonstrated by the lowest point on the medial condyle moving anteriorly at a greater rate than on the lateral side, implying a lateral pivot (Fig. 2A). If fluoroscopic data is compared across implants, the AP motion of the lowest femoral points observed in this study for the INNEX design is very similar to previous measurements in a PFC Sigma design, which also featured an abrupt radius change, but is specifically different from the more recent Attune design which has a gradual radius change and shows a continuous lateral roll-back^21,22^. Apparently, the differences in joint kinematics between the INNEX implant in this study and natural knee are likely to be due to the geometry of the tibia inlay, which constrains excessive posterior movement of the lateral condyle, leaving anterior movement on the medial side as the only option for further external rotation of the femur.

When the mean ML axial force distribution and pivot location are compared between the congruent and non-congruent flexion ranges, both are mostly medial near full extension, and more lateral in the non-congruent range (Fig. 2B). However, a closer look across flexion ranges reveals no consistent relationship between MPD and MFR across patients. In some patients (K1L, K8L), the lateralization of the pivot occurs abruptly around FLEX1, while the loading still remains mostly medial until higher flexion angles (Fig. 4). In these patients, the pivot location seems to be primarily determined by the activity (all subjects showed similar kinematic patterns), in addition to the ML distribution of the axial force, as well as other factors, probably including the limitation of AP motion by the slopes of the tibial inlay, subject specific implantation, and the tension of the surrounding soft tissue structures, including possible co-contraction^35^. The limitations of the AP motion by the congruent geometry could explain why the pivoting was more strongly related to the AFR than to the MFR in some cases (Table 3). While the investigated ML distribution of the axial TF load includes the axial components of the muscle and ligament forces, their shear components can affect the pivoting as well. The wide range of R^2^ values in the MPD~MFR correlation seems to indicate these shear forces from muscle and ligaments play a greater or lesser role in individual subjects, which could also explain the differing ranges of AP-forces between the patients (Fig. 4, Table 4).

It should be pointed out, that the observed AP-motion during loaded flexion does not necessarily imply the same AP-motion during the stance phase of walking, where the flexion range is smaller but the MFR range can be greater^4,7^. Another limitation of this study is the small number of patients and the specific characteristics (single, ultra-congruent design) of the implant. The high implant congruency likely reduces the range of the AP-motions and leads to a complex contact interaction which could not be fully investigated in this study, but potentially will be the focus of future finite element analyses. Despite these limitations, this is the largest cohort of patients with *in vivo* forces synchronously measured with fluoroscopic kinematics. With this, the subjects examined give a first impression on how measured TF forces and the combined fluoroscopic kinematics are related.

In conclusion, even with a symmetrical tibial inlay, the ML position of the pivot for the axial TF rotation was not determined by the ML distribution of the axial load alone. Especially when the posterior motion of the lateral condyle is restricted by the design, some patients shift the pivot laterally, even when the medial compartment still experiences a higher axial load. Further studies into the activities and factors driving TF kinematics, and their relationship with the pivot location are clearly required in order to better understand the interaction of kinematics and loading in the knee. Using fluoroscopic measurement techniques combined with the 3D analysis employed in this study may serve as an ideal tool to verify whether a specific kinematic goal of a new TKA design, is actually achieved *in vivo* and across mechanically loaded activities.

## Data Availability

Sample data that support the findings of this study is available on the CAMS-Knee-Project website (https://cams-knee.orthoload.com/data/data-download). Restrictions apply to the availability of the implant geometry data, which were used under license for the current study, and so are not publicly available.
