# Author Correction: Tibio-Femoral Contact Force Distribution is Not the Only Factor Governing Pivot Location after Total Knee Arthroplasty

**DOI:** 10.1038/s41598-019-41668-2

**Published:** 2019-04-11

**Authors:** A. Trepczynski, I. Kutzner, P. Schütz, J. Dymke, R. List, P. von Roth, P. Moewis, G. Bergmann, W. R. Taylor, G. N. Duda

**Affiliations:** 1 Julius Wolff Institute and Center for Musculoskeletal Surgery, Charité – Universitätsmedizin Berlin, corporate member of Freie Universität Berlin, Humboldt-Universität zu Berlin, and Berlin Institute of Health, Berlin, Germany; 2 Institute for Biomechanics, ETH Zürich, Zürich, Switzerland

Correction to: *Scientific Reports* 10.1038/s41598-018-37189-z, published online 17 January 2019

This Article contains a typographical error in the Materials and Methods section where,

“Hence, only a brief summary of the measurements is provided here: Six TKA patients: 5 male, 1 female, aged 77(65–80) [median, range] years, 5–7 y post-op, mass 88(63–95) kg, height 174(165–175) cm, implanted with a posterior cruciate sacrificing (PCS) INNEX-FIXUC implant (Zimmer, Switzerland), performed 5–6 repetitions of a squat activity (Fig. 1A)”.

should read:

“Hence, only a brief summary of the measurements is provided here: Six TKA patients: 5 male, 1 female, aged 77(65–80) [median, range] years, 5–7 y post-op, mass 93(67–101) kg, height 174(165–175) cm, implanted with a posterior cruciate sacrificing (PCS) INNEX-FIXUC implant (Zimmer, Switzerland), performed 5–6 repetitions of a squat activity (Fig. 1A)”.

